# Downstream Processing of Crude Ultrasound-Extracted Pectin From Saba Banana (*Musa acuminata* x *balbisiana* (BBB Group) “Saba”) Peel

**DOI:** 10.1155/2024/9892858

**Published:** 2024-08-17

**Authors:** Rachel S. Rodulfo, Katherine Ann T. Castillo-Israel, Prince Joseph V. Gaban, Ma. Cristina R. Ilano, Joshua B. Benedicto, Mark Anthony A. Badua, Joel P. Rivadeneira

**Affiliations:** ^1^ Institute of Food Science and Technology College of Agriculture and Food Science University of the Philippines Los Baños, Los Baños, Laguna 4031, Philippines; ^2^ National Institute of Molecular Biology and Biotechnology (BIOTECH) University of the Philippines Los Baños, Los Baños, Laguna 4031, Philippines

## Abstract

Ultrasound-assisted extraction of pectin from Saba banana (*Musa acuminata* x *balbisiana* (BBB Group) “Saba”) peels produced crude pectin that requires further purification. Two downstream processes (alcohol washing (AW) and acid demethylation (AD)) were compared. AW involved gelatinous precipitate washing with 85% alcohol and pressing to squeeze out liquids, while AD involved a sequential AW of the dried pectin with 60% acidified alcohol, and 60% and 95% alcohol solutions. Results showed that both methods produced low methoxyl pectins with similar color, yield, and moisture content, with no significant (*p* > 0.05) differences observed. AD, however, produced pectin with better quality in terms of ash content and anhydrouronic acid (AUA) content relative to the control. Fourier transform infrared spectra revealed that the samples contain -OH, C-H, COO^−^, COO, and C-O groups, but only AD has COO-R due to structural modification. Overall, AD has the potential to improve the quality of crude ultrasound-extracted pectin from Saba banana peels. Yet, pre-extraction processing methods are necessary to meet FAO color standards for pectin.

## 1. Introduction

Pectin is a heteropolysaccharide primarily composed of chains containing 300–1000 galacturonic acid (GalA) units linked by *α*-(1→4) bonds [1]. It is part of the primary cell walls in most plants, contributing to mechanical strength and flexibility through interactions with other cell wall components [2]. It plays a crucial role in gelling, thickening, and stabilizing products.

Pectin can be described by their anhydrouronic acid (AUA) content and degree of esterification (DE). The AUA content (which characterizes the GalA) is a measure of the purity of pectin [3]. A higher AUA value means that pectin has a higher purity. Higher purity results in a better gel strength [4]. For food applications, the AUA content of pectin must not be less than 65% [5]. On the other hand, the DE, which is also called the degree of methylation, describes the ratio of the moles of methyl ester to the moles of GalA. In terms of DE, pectin can be classified as low methoxyl pectin or high methoxyl pectin. Low methoxyl pectin are those with DE < 50% while high methoxyl pectin are those with DE > 50%. Mechanisms of gel formation differ based on whether the pectin is low methoxyl or high methoxyl. For low methoxyl pectin, gel formation is based on the “egg-box” model, which involves the crosslinking of the ionic carboxyl groups of the pectin in the presence of divalent cations, such as calcium ions. In contrast, the mechanism of gel formation of these high-methoxyl pectin is based on cross-linkages brought about by nonionic interactions, such as those that form in the presence of sugars and acids [6]. With different gelling mechanisms come different food applications. Low methoxyl pectin is often applied to low-sugar products and dairy products. The stability and texture of water-soluble soy extract can also be improved by low methoxyl pectin. On the other hand, high methoxyl pectin is often applied to jams, jellies, marmalades, sweets, and desserts [7].

During extraction, pectin is separated from other soluble components in the extract via ethanolic precipitation, resulting in a gelatinous precipitate with high impurity concentrations. Wang et al. [8] reported that the precipitate of crude grapefruit pectin was found to contain 10% glucose in the monosaccharide composition analysis, which requires purification to remove small molecules such as phenolics, flavonoids, and non-pectic polysaccharides that consist a substantial quantity of glucose. The insolubility of these impurities in alcohol and/or their binding to the precipitate contributes to their presence.

Crude pectin displays a brown-black color, a contrast to the standard pectin which varies from white to light brown [9]. Various research has linked this dark color of crude pectin to the presence of oxidized polyphenols [10], water-soluble pigments [11], and low molecular weight impurities [12]. Moreover, some have suggested that this dark coloration is a result of tissue damage and enzymatic browning leading to melanin biosynthesis. Previous studies have linked tyrosinases in banana peels to browning reactions, triggered by tissue damage [13–15]. It is widely accepted that mechanical damage to the banana peel, especially when exposed to high temperatures, leads to cellular damage and subsequently, a blackening of the banana peel. While pectin color varies by source, using dark-colored pectin in food could negatively impact the final product's color. Additionally, uniform incorporation into food matrices may be challenging, affecting product consistency.

Impurities in crude pectin not only affect its color but also compromise overall quality. When impurities reduce GalA content and DE below the minimum standard, it weakens gel-forming capacity and functionality, resulting in inconsistent gelling behavior and less stable gels. Ultimately, it affects the texture and quality of the final product. Moreover, if the DE is altered, the gelling mechanism of the pectin, and consequently its product applications, is changed.

Addressing crude pectin's impurities can be done by downstream processing. Downstream processing is the process of enhancing the quality of the product in terms of purity and concentration [16] which may include a series of unit processes [17].

In this study, the quality of the crude ultrasound-extracted Saba banana pectin was purified using two downstream processing methods: (1) washing the gelatinous precipitate with alcohol following ethanolic precipitation and (2) sequential washing of dried pectin powder using acidified alcohol and alcohol of varying concentrations. These methods were compared to recommend an efficient protocol for the downstream processing of crude pectin.

## 2. Materials and Methods

### 2.1. Materials

The crude ultrasound-extracted pectin (extracted from 800 g dried Saba peels) was obtained from the Institute of Food Science and Technology, University of the Philippines, Los Baños, Laguna, Philippines (IFST-UPLB). Various technical grade ethanol solutions (60%, 85%, and 95%) and analytical grade HCl were used for the downstream processes. Analytical grade reagents, including NaOH, HCl, and phenolphthalein indicator, were standardized for chemical analyses to ensure consistency and accuracy.

### 2.2. Downstream Processing of Pectin

#### 2.2.1. Control

The crude ultrasound-extracted pectin was dried to constant weight at 45°C.

#### 2.2.2. Alcohol Washing (AW)

The purified pectin was obtained following the method of Labrada et al. [12] with slight modifications. The crude ultrasound-extracted pectin was washed with 600 mL 85% ethanol and then dried to constant weight at 45°C.

#### 2.2.3. Acid Demethylation (AD)

The acid-alcohol insoluble pectin material was obtained following the method of US Pharmacopeia [18], with slight modifications. The crude ultrasound-extracted pectin was washed with 600 mL 85% ethanol and then dried to constant weight at 45°C. The dried pectin was mixed with the acidified alcohol (composed of concentrated hydrochloric acid and 60% ethanol with a ratio of 1:20 v v^−1^) at 1:21 m v^−1^ ratio and stirred constantly for 15 min. The mixture was allowed to settle and then transferred to a coarse-sintered glass filter connected to an oil-free vacuum pump (Rocker 300, Taiwan). The filter cake was then washed with an acidified alcohol solution (1:18 m v^−1^ ratio) followed by an equal volume of 60% ethanol. Lastly, the filter cake was washed with 95% ethanol (1:4 m v^−1^ ratio) and then dried to a constant weight at 45°C.

### 2.3. Pectin Yield

The yield was determined as the ratio of the weight of dry pectin to the original weight of dry Saba banana peel (800 g) prior to ultrasound-assisted extraction of pectin on a dry basis, considering Equation (1).

(1) $$\begin{array}{c} \text{Yield }\left(\%\right)=\frac{\text{Pectin dry weight }\left(\text{g}\right)}{800\text{ g}}\times 100 \end{array}$$

### 2.4. Physicochemical Characteristics of Pectin

#### 2.4.1. Color Measurement

The color parameters of the pectin samples were determined using a chromameter (CR-400, Konica Minolta Inc, Tokyo, Japan) by measuring *L*^∗^ (lightness), *a*^∗^ (red-green), and *b*^∗^ (yellow-blue) values in the International Commission on Illumination (CIE) system.

#### 2.4.2. Moisture and Ash Contents

The moisture and ash contents were determined following the standard method of AOAC [19] with slight modifications. Approximately 0.5 g of pectin sample was placed into a tared crucible. The sample was dried in an oven at 100 ± 5°C until constant weight and cooled in a desiccator. After that, the tared crucible containing the dried sample was placed in a muffle furnace for 5 h at 550 ± 5°C, cooled in a desiccator, and weighed. The moisture and ash contents were determined using Equation (2) and Equation (3), respectively.

(2) $$\begin{array}{c} \text{Moisture content }\left(\%\right)=\frac{\text{Dried sample},\text{g}}{\text{Pectin weight},\text{g}}\times 100 \end{array}$$

(3) $$\begin{array}{c} \text{Ash content }\left(\%\right)=\frac{\text{Ash weight},\text{g}}{\text{Pectin weight},\text{g}}\times 100 \end{array}$$

#### 2.4.3. AUA Content and DE

The AUA of the pectin was determined using a modified method based on Ranganna [20] and Wathoni et al. [21]. A 0.5 g pectin sample in a 250-mL flask was moistened with 2 mL of 95% ethanol. Sodium chloride (1 g), carbon dioxide-free water (100 mL), and phenolphthalein indicator (six drops) were added. The mixture was titrated with 0.1 N NaOH until the pink end point (initial titer, *V*_*I*_). Next, the solution was combined with 25 mL of 0.25 N NaOH, shaken, and left for 30 min at 25°C in a closed Erlenmeyer flask. Afterward, 25 mL of 0.25 N HCl and the phenolphthalein indicator were added, and then titrated with 0.1 N NaOH (saponification titer, *V*_*S*_). The percentages of AUA and the DE were calculated according to Equation (4) [20] and Equation (5) [18], respectively.

(4) $$\begin{array}{c} \text{AUA }\left(\%\right)=\frac{176\times 0.1V_{S}\times 100}{w\times 1000}+\frac{176\times 0.1V_{I}\times 100\;}{w\times 1000} \end{array}$$

(5) $$\begin{array}{c} \text{DE }\left(\%\right)=\left(\frac{V_{S}}{V_{I}+V_{S}}\right)\times 100 \end{array}$$

### 2.5. Fourier Transform Infrared Spectroscopy (FTIR)

The spectra of the pectin samples were generated in an FTIR spectrometer with wavenumbers ranging from 400 to 4000 cm^−1^ in attenuated total reflection mode at a resolution of 4 cm^−1^ with 64 scans.

### 2.6. Statistical Analysis

All experiments were performed in duplicate, and the results were expressed as mean ± SD. Analysis of variance (ANOVA) and Tukey's post hoc test (*p* < 0.05) were determined using Jamovi version 2.3 (The Jamovi Project, 2022, Sydney, Australia).

## 3. Results and Discussion

### 3.1. Yield

The yields of the control (2.81 ± 0.62%), AW (1.79 ± 0.12%), and AD (1.3 5 ± 0.05%) were not significantly different (*p* > 0.05) from each other, irrespective of the downstream processing method used. However, these yield values were lower than the 16.54% reported by Castillo-Israel et al. [22] for conventionally extracted unripe Saba banana pectin. The lower yield could be attributed to the differences in the extraction, isolation, and purification conditions used.

### 3.2. Physicochemical Properties

The physicochemical properties of the purified pectin are shown in Table 1.

#### 3.2.1. Color

**Figure 1:**
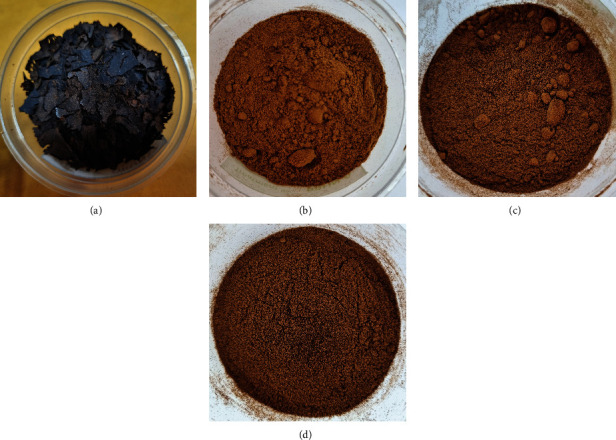
Appearance of ultrasound-extracted pectin using different downstream processing methods. (a) Crude ultrasound-extracted pectin flakes. (b) Control. (c) Alcohol-washed pectin. (d) Acid-demethylated pectin.

All pectin samples had an initial brown-black color and hard, brittle texture after drying, but transitioned to a yellowish-brown color after undergoing grinding and sieving processes (Figure 1). Comparable results were reported with pectins extracted from lemon-mango peel [23], mango peel [12], South African prickly pear peel [24], and dragon fruit peel [25]. The application of AW to the crude pectin led to a significant (*p* < 0.05) color alteration compared to the control, transitioning from a yellowish-brown to dark brown. This was in contrast with the results reported by Labrada, Lobarbio, and Taboada [12]. They found that after extensive washing with absolute ethanol, including mashing and pressing, the color of the crude mango pectin transformed from a dark brown, hard, brittle structure to a lighter brown and soft, cotton-like texture. Further purification using the AD method showed no significant improvement in the pectin's color.

The color parameters of AW pectin and AD pectin diverged from those reported by Khamsucharit et al. [2], where the lightness (*L*^∗^), redness (*a*^∗^), and yellowness (*b*^∗^) of pectin extracted from various banana peels ranged from 77.33 to 85.30, 2.09–3.88, and 5.37–8.59, respectively. As previously noted, the dark color of Saba banana pectin can be attributed to the presence of oxidized polyphenols [10], water-soluble pigments [11], and low molecular weight impurities [12]. While AD managed to remove a fraction of the total phenolics, it did not significantly enhance the color, suggesting that some phenolic compounds may be bound to pectin molecules. This suggests that these particular compounds could potentially withstand extraction processes such as acid hydrolysis and/or successive ethanol washes. Given the FAO [9] standards for acceptable pectin colors, which include white, yellowish, light greyish, or light brownish, neither AW pectin nor AD pectin passed the color test. This could adversely impact consumer preference as the dark color of pectin might be equated with inferior quality.

Dark pectin, as an ingredient in food processing, poses several issues. It can affect the product's color and its stability during storage, which may be undesirable for light-colored foods like jams or jellies. Moreover, its phenolic compounds may occasionally create a slightly bitter taste. Furthermore, achieving uniform incorporation into food matrices is challenging, affecting overall consistency. Therefore, alternative methods might be necessary to remove the dark color of pectin.

#### 3.2.2. Moisture Content

The moisture content of all samples adhered to the FAO [9] standard for commercial pectin, which is less than 12%, signifying that all samples were properly dried. The low moisture content, coupled with a water activity below 0.8, will further inhibit the growth of microorganisms that could potentially compromise the quality of the pectin.

#### 3.2.3. Ash Content

The ash contents in all samples were significantly (*p* < 0.05) different. The control sample had an ash content of 14.36 ± 0.06%, which reduced to 11.07 ± 0.09% after AW, and further declined to 1.79 ± 0.00% after AD. This indicates that the acid hydrolysis process effectively extracted the bound alcohol-insoluble impurities, thereby enhancing the quality of pectin. As suggested by Castillo-Israel et al. [22] and Konrade et al. [26], the upper limit for ash content to form a good-quality gel from pectin is 10%. Therefore, the AD method demonstrates the potential in producing a good-quality pectin gel.

#### 3.2.4. DE

The DE of AD pectin (46.23 ± 0.44%) was found to be significantly (*p* < 0.05) lower when compared to both the control (48.65 ± 0.14%) and AW (49.79 ± 0.07%) pectin samples. This is in contrast to the higher DE values reported in previous studies by Khamsucharit et al. [2] and Castillo-Israel et al. [22], which ranged from 63.15 to 72.03% and 75.03%, respectively. The variance in DE can be linked to the alterations in the pectin's structure during the downstream processing, which includes a reduction in methyl ester groups and an increase in GalA content. Regardless, the DE for all samples remained below 50%, classifying them as low methoxyl pectins according to the standard definition of FAO [9]. This suggests that the samples are likely to form a gel in the presence of calcium ions within a pH range of 2–6, independent of their sugar content [27].

#### 3.2.5. AUA

The control sample had an AUA content of 57.05 ± 0.23%. Subsequently, the purity increased to 61.66 ± 2.16% after applying AW to the gelatinous precipitate, albeit without statistical significance. Notably, this surpasses the 39.68% AUA content found in conventionally extracted unripe Saba banana pectin, as reported by Castillo-Israel et al. [22]. The increase in AUA content in the crude pectin following AW can be attributed to the removal of alcohol-soluble phenolic compounds, which were present in high concentrations in the control sample [28]. However, AW's pectin purity remains below the 65% minimum AUA content required for commercial-grade pectin [9]. Alam et al. [29] noted that no single solvent can extract all forms of phenolic compounds due to differences in their polarity, size, and attachment to the crude extract. Moreover, simple solvents like ethanol are incapable of extracting, esterifying, or glycosylating phenolic compounds that are bound to the crude extracts and require treatment with weak alkali and/or acids. Dong and Yao [30] and Pralea et al. [31] proposed that phenolic compounds can be extracted through acid hydrolysis or successive washing with organic solvents, such as ethanol.

When the crude pectin underwent the AD process, the AUA content significantly (*p* < 0.05) increased to 81.31 ± 0.50%. This increase meets the standards of both the FAO [9] and the US Pharmacopeia [18], which require a minimum AUA content of 65% and 74%, respectively, for commercial- and pharmaceutical-grade pectin. Therefore, it is acceptable for use in both food and pharmaceutical applications. This result demonstrates that the AD method is effective in removing a significant amount of non-uronide materials. Furthermore, AD could be an alternative purification method for producing low methoxyl pectin.

### 3.3. FTIR

**Figure 2:**
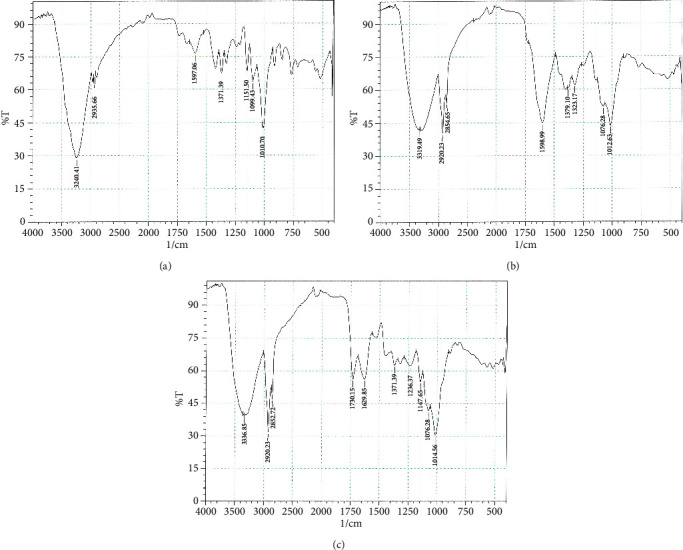
FT-IR spectra of pectin. (a) Standard. (b) Alcohol-washed pectin. (c) Acid-demethylated pectin.

Figure 2 and Table 2 present the infrared spectra of pectin processed through two distinct downstream processing methods, including a standard. The spectra revealed that all pectin samples exhibited absorption peaks within the ranges of 3336.85–3240.41 cm^−1^, 2935.66–2920.23 cm^−1^, and 1149.57–1076.28 cm^−1^. These peaks are indicative of the polysaccharides' characteristic absorption and are ascribed to the stretching vibrations of hydroxyl groups (O-H), the C-H bonds of CH_2_ and CH_3_ groups, and the C-O bonds, respectively. Additionally, absorption bands were observed around 1629.85–1597.06 cm^−1^, corresponding to the C=O stretching vibration of free carboxyl groups (COO^−^). Notably, the AD pectin uniquely exhibited an absorption peak at 1730.15 cm^−1^, which is attributed to the C=O stretching vibration of methyl-esterified carboxyl groups (COO-R). This suggests that the downstream processing methods used exert minimal impact on the pectin's chemical structure.

## 4. Conclusions

Both AW and AD had minimal effects on pectin yield but resulted in darker-colored pectin compared to standard commercial products. Crucially, both methods successfully lowered the moisture content to levels acceptable by the FAO (less than 12%), which is essential for maintaining pectin stability during storage and processing. Notably, AD outperformed AW in reducing ash content, contributing to higher AUA content—a desirable trait for various applications. The pectin produced through both methods was classified as low methoxyl, with AD pectin conforming to commercial and pharmaceutical-grade standards. Additionally, Fourier infrared transform spectra indicated minimal structural and branching pattern changes, suggesting that both AW and AD preserve pectin integrity. By enhancing overall purity, AD improves functionality and expands pectin's applicability across various industries. Therefore, AD is the preferred downstream process for enhancing pectin quality due to its efficient ash reduction, increased AUA concentration, low methoxyl classification, and minimal impact on pectin structure.

Further investigation is encouraged into the incorporation of the AD method during the purification stage of ultrasound-assisted extraction of pectin, in conjunction with ethanolic precipitation. This approach could potentially remove the dark coloration without compromising the structural integrity, functionality, gel-forming ability, and other properties of pectin. Moreover, it is suggested that melanin, present in the raw Saba banana peel, should be extracted during the pretreatment phase before the ultrasound-assisted extraction of pectin. This could also enhance the color quality of the extracted pectin. Lastly, identifying the specific impurities in the precipitates during pectin extraction could be investigated to establish a more appropriate method for pectin isolation.

## Figures and Tables

**Table 1:** Physicochemical properties of the crude and purified Saba banana pectin.

**Property**	**Control**	**AW pectin**	**AD pectin**
Color: *L*^∗^	51.89 ± 1.09^a^	43.64 ± 1.02^bc^	41.58 ± 0.22^bc^
Color: *a*^∗^	5.71 ± 0.17^a^	6.58 ± 0.25^bc^	6.89 ± 0.13^bc^
Color: *b*^∗^	13.53 ± 0.33^a^	11.54 ± 0.58^bc^	11.62 ± 0.34^bc^
Moisture content (%)	7.48 ± 0.11^a^	8.27 ± 0.11^bc^	8.16 ± 0.01^bc^
Ash content (%)	14.36 ± 0.06^a^	11.07 ± 0.09^b^	1.79 ± 0.00^c^
Degree of esterification (%)	48.65 ± 0.14^a^	49.79 ± 0.07^b^	46.23 ± 0.44^c^
Anhydrouronic acid (%)	57.05 ± 0.23^ab^	61.66 ± 2.16^ab^	81.31 ± 0.50^c^

*Note:* Values are presented as mean ± standard deviation. Different lowercase letters within the same row represent statistically significant differences at 5% significance (*p* ≤ 0.05) based on the Tukey post hoc test; control = dried ultrasound-extracted pectin from IFST-UPLB.

Abbreviations: AD = acid-demethylated pectin, AW = alcohol-washed pectin.

**Table 2:** Summary of the interpretation of the FTIR graph of pectin.

**Peak region (cm** ^**−1**^ **)**			**Group**	**Compound class**	**Reference**
**Standard**	**AW**	**AD**			
3240.41 3244.27	3319.49 3302.13	3336.85 3329.14	O-H	Hydroxyl compounds	[8, 26, 32–37]
2935.66	2920.23	2920.23	C-H	-CH_2_ groups,	[8, 38, 39]
—	2854.65	2852.72	C-H	CH_3_ groups	[34]
—	—	1730.15	COO-R	Esterified carboxyl groups	[8, 32, 34–38]
1597.06	1598.99	1629.85	COO^−^	Free carboxyl groups	[8, 32, 34–38]
1371.39	1379.10	1371.39	O-H COO	Hydroxyl compounds Carbonyl group	[33] [35]
1149.57 1012.63	1076.28 1012.63	1147.65 1014.56	C-O	Monosaccharide (pyranose and furanose)	[8, 33, 38]

## Data Availability

The data used to support the findings of this study are available from the corresponding author upon request.
